# Observations on Phlegmatia Dolens

**Published:** 1801-08

**Authors:** 


### *Observations on Phlegmatia Dolens, by* Dr. Hull.

[ Continued from our laſt, pp. 51—63. ]

### II. *Observations on Mr. White's Theory of the Disease.*

IN the ſecond part of his "*Inquiry*," the ingenious author makes the following remarks: "When a healthful woman, who has not had the *anasarca gravidarum*, is ſeized with this diſorder, in her lying-in in its ſimple, perfect, but uncomplicated plicated ſtate, and it is confined to one ſide *only*, which happens about nine times out of ten, it is then allowed by *all* authors who have written profeſſedly on the ſubject, that the ſwelling is confined to the limb, nates, lumbar and hypogaſtric region, groin, and labium pudendi of that ſide, and *I* muſt add, that it occupies all thoſe parts, and never in the ſmalleſt degree deviates from that line." p. 58.

Though it is clear from the former part of my paper, that the above ſtatement is incorrect, I am willing to admit on Mr. White's authority, that the affection was exactly of this kind in every caſe wherein he will ſay, that on a careful examination he found it ſo. Moreover, in diſcuſſing his theory of the diſeaſe, which he ſeems to deem applicable only to this particular variety, named by him *Phlegmatia alba dolens puerperarum*, I will not avail myſelf of the objections that might be urged againſt it, from taking a more comprehenſive view of the malady; but will endeavour to prove, that his theory is at variance with his own obſervations and caſes. His eighth caſe I muſt beg leave to inſert entire in this place, as I ſhall have occaſion to refer to different paſſages in it hereafter; and I am deſirous that it ſhould appear to every reader, that the quotations from it are made with perfect fairneſs.

"Elizabeth Rothwell, of Gravel Lane, Salford, a very healthful young woman, aged 24, free from any chronic diſorder, and who had always enjoyed a moſt perfect ſtate of health, was delivered of her ſecond child, as ſhe lay upon her left ſide, on the 19th of January, 1783, by a midwife; *had an easy time*, and ſuckled her child. She was kept very warm, never went out of her room, nor did any thing by which ſhe could poſſibly catch cold, till twenty days after delivery, when a window was opened in the room about a minute or two. *In an hour after, she was seized with a pain in her right side and shoulder*; *but had no cough*; *she was bled, which eased the pain*. Two days after, ſhe was attacked with pain in the groin, labium pudendi, thigh and leg of the ſame ſide, *which swelled, and then the pain abated.* The parts were tenſe and hard, *attended with fever.* She was made a home patient of the Infirmary under the care of Dr. Bell, and is now got perfectly well." *Inquiry*, P. I. p. 23 and 24.

Before I enter upon the examination of the theory of this diſeaſe, as given by Mr. White, it may not be improper to premiſe a few obſervations. 1. On the nature of the fluid poured out by the exhalants into the different circumſcribed cavities of the human body, both in a healthy and diſeaſed ſtate. 2. On the nature and properties of the lymph. And, 3. On the ſtructure, courſe, &c. of the lymphatics, eſpecially in in the inferior extremities and the other parts of the trunk, usually affected in Phlegmatia Dolens.

1. The *exhaling fluid* in the natural state of the body, is not in sufficient quantity to distend the cavities into which it is constantly passing from the extremities of the arteries opening upon them; it merely moistens the internal surfaces of the membranes; to the touch it is viscid and mucilaginous, and is analogous to the serum of the blood. In dropsical cases it is accumulated in sufficient quantity to be easily obtained and subjected to a chemical analysis. M. Ronelle and Fourcroy have examined the exhaled fluid of hydropics, as taken from the chest, pericardium, ovary, abdomen, &c. and have found it possessing all the properties of the serum. According to the latter author, it is ordinarily viscid, yellowish, of a sweetish and saltish taste; it sometimes contains greyish yellow flakes in greater or smaller quantity, which consist of coagulated albumen. It coagulates by heat, acids and alcohol, precipitates on the addition of calcareous and metallic salts, and turns the syrup of violets green. The coagulum, formed by heating it, is of a sulphur colour, is light, porous, and of a tremulous consistence; a slightly yellow liquid oozes from it, which is not coagulable, and contains sulphur, phosphates of lime, &c. Diluted with water and heated, the exhaled fluid of hydropics affords a milky liquor, which does not coagulate, but yields a yellow pellicle: A true jelly cannot be obtained from it, therefore the albuminous matter contained in it, is not in the true state of gelatine.* In inflammatory diseases the exhaled fluid thrown into the different cavities of the body, coagulates, a portion of the coagulating lymph of the blood, or *fibrine*, according to the new chemical nomenclature, being contained in it. A separation of the fibrine from the serous part afterwards takes place, and this, when it becomes perfectly solid, either attaches itself wholly to the internal surfaces of the membranes lining these cavities, or partly adheres to them, and partly is formed into flakes or loose coagula, and intermixed with the fluid part.

2. The *lymph*, or fluid, contained in those absorbents which do not convey chyle to the thoracic duct, has by different writers been considered as the same as, or at least as analogous to, the serum of the blood; and *a priori* it may seem allowable to consider it as the same identical fluid with that exhaled into the circumscribed cavities, or with this and a slight admixture

* See Fourcroy Systeme des Connaissances Chimiques, 8vo. Tom. ix. p. 165.

of the other fecreted fluids, according to the fituation of the lymphatics. The properties of the lymph, however, have not been fufficiently determined, owing to the difficulty of obtaining it pure, and in fufficient quantity for a chemical analyfis. It is pretty well afcertained, that its properties do vary occafionally. Soemmerring mentions the cafe of a healthy woman, in whom the lymphatics of the foot were varicofe, and lymph flowed out freely on pricking them with a needle.* This lymph was found to have the following properties: It was clear and pellucid, of a flightly yellow colour: Its tafte was bland, and faline (muriatic). Expofed to the air, of the temperature of 50° of Fahrenheit's thermometer, for feveral hours, its fluidity was fcarcely diminifhed. But on the addition of alkohol and the mineral acids it became turbid, and after fome hours the precipitate fell to the bottom, and the more fluid part fwam at the top. When it was evaporated fpontaneoufly in open veffels, or in a gentle heat, a good deal of a yellow tenacious matter, refembling gum arabic, and cracking like it, and pellucid as amber, remained with fome flender faline cryftals depofited upon it. When evaporated to one half the original quantity over the fire, it affumed a gelatinous nature. Being placed in 50° of Fahrenheit, it did not eafily putrefy; but after fome weeks became turbid, emitted a cadaverous odour, and was changed into an apparently puriform matter. Muriate of mercury, added in powder, imparted to it an opaline opacity, and a variegated reddifh colour, and preferved it from putrefaction. Pieces of camphor alfo prevented its putrefaction, infomuch that, after all the camphor had difappeared, the lymph, which had indeed been diminifhed by exhalation, remained for fome time liquid and pellucid. Peruvian bark in powder did not long prevent it from putrefying; it was found inferior in this refpect to lime-water. This is by much the moft circumftantial account of the properties of the human lymph which I have been able to meet with. Mr. Cruikfhank fays, in the pureft ftate that he could procure the lymph, "it refembled water in fluidity, was tranfparent, fometimes of a ftraw colour, or even brown; it alfo either coagulated wholly on extravafation, or coagulated in part, as the animal from which it was taken was either ftronger or weaker. The fame thing happened in the dead body, where it coagulated from reft, or in confequence of death;" whence he fufpects, that the fluid found in the lymphatics is in part the coagulable lymph of the blood.†

Mr.

* De Corporis Humani Fabrica, Tom. v. p. 416 and 420.

† Anatomy, &c. p. 96. See alfo Hewfon's Exp. Inq. P. ii. p. 238.

Mr. Aſtley Cooper informs me, that in an experiment upon the abſorbents of a horſe, he put a watch glaſs under the divided abſorbents, and caught the lymph as it iſſued, which coagulated in about three minutes, and that he has alſo taken lymph in the ſame way from the abſorbents of the neck of a dog, and found that it coagulated. From what has been already ſtated, it appears that the lymph varies in its diſpoſition to coagulate; and from an intereſting caſe, related by Mr. Patch, it would ſeem that it ſometimes aſſumes a milky appearance. The boy, who was the object of this caſe, had a milky diſcharge in conſiderable quantity from a ſmall orifice in the groin. Three or four ſpoonsful of the fluid were brought to him, which, he ſays, "appeared like ſcalded milk; and ſome of it being heated over a candle, it ſoon turned to a ſoft curd.* There is reaſon to believe, that this fluid was diſcharged from the inguinal abſorbents, and that there was an admixture of adeps in it, as it was white, and the boy is ſtated to have been very much weakened by this evacuation. It is to be hoped, that thoſe practitioners, who have an opportunity of collecting the lymph from wounds, or ulcers of the groin, cubit, &c. will analyſe it with more care than has been hitherto done.

Fourcroy ſeems to think, that, by paying attention to this kind of reſearches, the abſorbents may be opened at pleaſure, and that this ſort of operation, which he names *lymphée*, may fulfil ſome uſeful indications; that it may remove lymphatic plethora, diminiſh the maſs of ſuperabundant white and nouriſhing juices, and evacuate the lymph that is accumulated in certain cavities of the body; for, ſays he, the depletion of a large lymphatic veſſel, and the void which ſucceeds it, muſt augment the force of ſuction and abſorption in one region, and ultimately in the whole of the abſorbent ſyſtem.† It does not appear that the lymph has ever jellied, or coagulated in the veſſels of the extremities in a living animal.

3. The *lymphatics* anaſtomoſe more frequently than the arteries, and the anaſtomoſing branches are in many parts of large ſize; from their very frequent communications with each other, they often form an irregular net-work.‡ Their coats are thinner

 and

* Edinburgh Medical Eſſays, Vol. v. p. 238.

† Syſteme des Conn. Chimiques, Tome ix. p. 169.

‡ "This *anastomosis* takes place not only between the ſmaller branches, but between the larger trunks and the glands themſelves."—"From one lymphatic on the top of the foot, a conſiderable number of other lymphatics on the leg and thigh, the greater number of the glands in the groin, on the edge of the pelvis, on the vertebræ, and the thoracic duct itſelf, may in and more tranſparent, but ſtronger than thoſe of the red veins, whence they are enabled to ſupport a column of mercury of conſiderable length; when diſtended, they have a jointed appearance, owing to their valves. In the inferior extremities, the lymphatics are in two general ſets, or layers, one ſuperficial, the other deep-ſeated. The ſuperficial lymphatics are very numerous; compared with the ſubcutaneous veins, they are, according to Mr. Cruikſhank, as fourteen to one. They accompany the veins, named *V. saphena major et minor.* The deep-ſeated lymphatics accompany the large blood-veſſels, and are leſs numerous than the ſuperficial ones, but are at leaſt double the number of the arteries. Both theſe ſets of lymphatics enter the inguinal glands. Part of the lymphatics of the external parts of generation in women, of the lymphatics of the inferior part of the abdomen, of the loins, nates, and vicinity of the anus, paſſes alſo into the inguinal glands; whilſt another part of them paſſes by the neareſt road into the pelvis, avoiding the inguinal glands: The lymphatics of the right and left ſides of the inferior part of the trunk anaſtomoſe with each other; ſo that the lymphatics of one lower extremity, and of the other parts of the trunk, uſually affected in Phlegmatia Dolens, when confined to one ſide, are not more diſtinct than the blood-veſſels of the ſame parts are.* Of the lymphatics, which proceed from the inguinal glands, and paſs under Poupart's ligament, ſome accompany the external iliac artery; others deſcend along the ſide of the pelvis near the internal iliac blood-veſſels, and paſs through ſome of the conglobate glands ſituated there. With reſpect to the lymphatics which paſs through the inguinal glands, it may not be improper to remark here, that theſe glands are the centre or focus, and that the lymphatics of the loins and abdomen may with as much reaſon be conſidered as the extreme points of this part of the lymphatic ſyſtem, as the lymphatics of the leg and foot. Now we find, that in five or ſix of the fourteen caſes related by Mr. White, the diſeaſe in queſtion began in the back, and extended or deſcended to the groin, &c.† In theſe caſes, therefore, the complaint, if it began in the lymphatics, according to Mr. White's hypotheſis, muſt have begun at a diſtant or extreme point of the affected part of the lymphatic ſyſtem; that in the ſame manner be injected."—"The intention of Nature by theſe anaſtomoſes, is evidently *to secure a number of roads*, by which theſe important fluids, the chyle and lymph, may be carried into the blood." *Cruikſhank's Anat. of the absorbing Vessels of the Human Body*, p. 85.

* See Mr. White's Inquiry, Part ii. p. 58.

† See Mr. White's Inquiry, Part i. Caſes 1, 2, 3, 4, 9, and 12.

that is, at a confiderable diftance from the groin or brim of the pelvis, if we follow the route of thefe lymphatics. Why then, agreeably to the fame hypothefis, may not the complaint begin in a diftant part of the inferior extremity, as in the lower part of the thigh, or in the leg, and afcend or extend to the groin? And upon what ground can thofe cafes be rejected, which begin in the leg, and are attended with the fame fymptoms as thofe which begin in the groin?

I will now proceed to examine the whole of Mr. White's Theory of *Phlegmatia alba dolens puerperarum*, as recapitulated in p. 125—131 of the Second Part of his Inquiry, premifing, that he has changed his ground, as far as relates to the manner in which the fuppofed injury of the abforbents is effected.

"When the brim of the pelvis," fays this very refpectable writer, "forms a prominent line on the body of the os pubis, and is as fharp as an ivory paper folder, or as fome knives, and jagged like a faw, and the gravid uterus, by the violence of the labour pains, forces the lymphatics againft this fharp edge, it muft cut or lacerate thofe lymphatic veffels which wrap round it and dip down into the pelvis, and they will difcharge their contents." From this quotation it appears, that the divifion of the lymphatics by a fharp prominent line* on the body of the os pubis is made a *causa*, *sine qua non*, of the difeafe. This fharp ridge is, I believe, a very rare occurrence, and Mr. White has not been able to fhew, that the lymphatics, which pafs over it into the tubular part of the pelvis, have in any cafe whatever been cut through by this ridge, or procefs. He affirms, indeed, in p. 99, that it cannot be denied, "that the fame fharp bone, which cuts through and lacerates the uterus and peritonæum, will alfo cut through or lacerate *all* the intermediate foft parts which lie betwixt the uterus and that fharp bone." Neverthelefs, I am of opinion, that neither the lymphatics paffing over this fharp bone, nor the periofteum which covers it, would neceffarily be divided in thofe cafes where the uterus or vagina is fo far injured as to give way during labour. Further, he has not adduced one fingle inftance to fhew, that this fharp ridge of the os pubis has ever exifted in the pelvis of any woman who has fuffered an attack of Phlegmatia alba dolens puerperarum: But he has circumftantially related the cafe of Jane Kinnerly in page 93, which goes as far, perhaps, towards proving the *contrary* of his hypothefis, as any one cafe can go to prove a general doctrine. This poor unfortunate woman's

* The ferrated appearance of this ridge, which Mr. White fpeaks of, I fhould apprehend, is produced after the periofteum has been feparated by maceration from fome injury.

woman's pelvis had actually the very ſharp prominent line on the body of the os pubis, which Mr. White makes a neceſſary circumſtance; and in conſequence thereof the uterus was cut, or torn through, in her eighth labour, and occaſioned her death. But, though ſhe had previouſly born ſeven children, ſhe never had the diſeaſe in queſtion in any of her lyings-in. Mr. White informs us, on the authority of Mr. Cruikſhank, that Mr. Hunter ſaw a caſe, in which the ſkin being pricked by a needle which had never been uſed before, or touched any infectious or irritating ſubſtance, occaſioned the lymphatics of the arm to inflame, and ſhew themſelves in the form of red lines, running towards the axilla." (p. 24.) Now, if ſo ſlight an injury as this can excite inflammation in the lymphatics, we may *a fortiori* ſuppoſe, that inflammation of the lymphatics would be excited in ſome inſtances at leaſt, if theſe were lacerated or divided, in conſequence of being violently compreſſed betwixt the child's head and the prominent line of the os pubis. Yet Mr. W. denies that this ever takes place in Phlegmatia alba dolens puerperarum. He ſays, "I believe there is not a ſurgeon in any practice in the kingdom, who has not ſeen both lymphatic glands and veſſels inflamed; and yet I will venture to ſay, that none of them ever ſaw the diſorder in queſtion occaſioned by, or even attended with, any ſuch ſymptoms." p. 26. Beſides, I have had under my care wounds of the lymphatics; and neither in theſe caſes, nor in any that I have read, not even in the caſe related by Mr. Patch, where the diſcharge was very conſiderable, does it appear that any intumeſcence, ſimilar to Phlegmatia dolens, ſupervened to their healing. And this diſeaſe alſo occurs to women after the moſt eaſy natural labours. From all theſe circumſtances I infer, that an injury of the lymphatics is not the remote cauſe of the diſeaſe, as is aſſumed by Mr. White.

We will now ſuppoſe that the lymphatics, which deſcend into the tubular part of the pelvis, are divided, and ſee whether Mr. White's theory, with this conceſſion, will account for the phænomena of the diſeaſe in a ſatisfactory manner. I am decidedly of opinion that it will not.

He next ſays, "In ſome caſes the extravaſated lymph will be immediately abſorbed by the lymphatics in the neighbourhood. In others it will accumulate, coagulate, and give pain, ſome days prior to the ſwelling of the limb, by ſeparating the peritonæum from its connections with the adjacent parts, and at laſt will be abſorbed. But, in ſome few caſes, it may not be abſorbed, but *produce an abſceſs*." The only obſervations which I ſhall make upon this extract, are, 1. That in the former part of his *Inquiry*, &c. p. 38, where he is informing us us that this diſeaſe is not a phlegmon or an eryſipelas, he adds, "neither is it an iliac abſceſs, or an abſceſs under the faſcia lata, *as it never comes to ſuppuration*;" which is directly contradictory of the paſſage juſt quoted. 2. That Mr. White ſays, in the ſecond part of his *Inquiry*, &c. p. 8, he is "well ſatiſfied that there is not one" (caſe) "of the genuine diſeaſe, in its ſimple, uncomplicated ſtate, ſufficiently authenticated, that ever proved fatal; not one that was ever attended with any external inflammation, abſceſs, gangrene, or burſting of the ſkin in the legs or thighs, as in anaſarca." Yet Mr. W. tells us, in another part of the ſame work, p. 80, that "whenever the ſwelling of the limb loſes its elaſticity, and pits upon preſſure, it is probable that many of the lymphatics burſt." Now, if this be the caſe, why may not the extravaſated lymph in the leg, or thigh, produce an abſceſs here as well as within the cavity of the pelvis; an inſtance of which Mr. W. mentions as having actually occurred in the caſe of Jane Waters. He may not have ſeen a caſe where a ſuppuration of the limb took place, but it cannot be ſuppoſed that he has ſeen every termination of this diſeaſe; and there is but too much evidence to prove, that this troubleſome complaint has taken place in various inſtances.*

He then proceeds to ſay, "In a ſpace of time, generally betwixt twenty-four hours and ſix weeks, the orifices in the ruptured lymphatics will cloſe, and they will be gorged with lymph, which will be impeded in them, but it will continue to flow in thoſe which have not been ruptured, particularly in the deep-ſeated lymphatics which accompany the iliac artery, and, *by anaſtomoſing with thoſe which have been ruptured, will prevent any material injury for the preſent, and in time will entirely ſupply their place.* Mr. W. has not ſufficiently explained himſelf upon the ſtate of the divided extremities of the lymphatics, betwixt the time of receiving the injury and the commencement of the diſeaſe. If the divided extremities cloſe quickly, the diſeaſe, if it be owing to this circumſtance, ſhould ariſe much ſooner than is generally found to be the caſe. If they be gradually contracting for a fortnight or more, the anaſtomoſing branches being gradually ſuffering a dilatation during this period, ſhould totally prevent, I think, any ſwelling of the limb from taking place at the time the wounded lymphatics completely cloſe. Again, if, as Mr. W. has aſſerted in the above quotation, the anaſtomoſes of the injured with the entire lymphatics,

* The authority of Calliſen, for which Mr. White has a high reſpect, may be here adduced, "Interdum œdema puerperarum in abſceſſum lacteum vel lymphaticum tranſit." *Princ.* P. II. §. 39.

phatics, will, *for the preſent, prevent any material injury*, it may be confidently expected that they will effectually prevent all injury afterwards. From conſidering the conſequences of tying the large blood-veſſels of an inferior or ſuperior extremity, and from viewing the number and courſe of the lymphatics of a lower extremity,† I am induced to believe, that if every lymphatic which paſſes over the ridge of the os pubis down into the pelvis were tied, or obſtructed, ſo that a ſingle drop of lymph could not be tranſmitted through them, the remaining ſound lymphatics would, by means of their frequent and large anaſtomoſes with the obſtructed ones, eaſily convey the whole of the lymph to the thoracic duct, ſo that, at moſt, only a comparatively ſlight and gradual tumefaction of the limb, unattended with the violent pain and other ſymptoms of Phlegmatia dolens, would be produced. The ſuppoſed cauſe therefore appears to me to be totally inadequate to the production of the diſeaſe.

"By the obſtruction of the lymph," continues Mr. White, "the groin, labium pudendi, and upper part of the thigh ſwell, the tumour gradually extends towards the leg and foot, and grows very painful, white, tenſe, elaſtic, hard, gloſſy, and uniform." There is no part of Mr. White's theory which ſurprizes me ſo much, as his conſidering the tumefaction, during the firſt ſtage of the diſeaſe at leaſt, as ariſing ſolely from the diſtenſion of the lymphatics. This ſuppoſition is, in my opinion, wholly untenable, and, as far as I know, is only maintained by Aſtruc and Mr. White. The progreſs and uniformity of the ſwelling, all militate ſtrongly againſt it. In the firſt caſe of his *Inquiry*, &c. Part I. p. 13, our Author ſays, that the progreſs of the ſwelling was "ſo quick, that the whole limb was, in the courſe of twenty-four hours, diſtended to *twice its natural ſize*;" and, in the ſixth caſe, p. 21, he ſays, "the pain deſcended into the groin, labium pudendi, thigh and leg of the ſame ſide, which ſwelled to *three or four times the natural ſize*." An inferior extremity of moderate ſize, for example, one 36 inches long and 4 inches in its mean diameter, is equal to 452 cubic inches, or about 15½ wine pints; conſequently, if a limb of this ſize be diſtended to twice its natural magnitude, that is, till it contain twice as much matter, within twenty-four hours after the attack of this diſeaſe, 15½ wine pints of matter muſt be added to the limb in that ſhort ſpace of time; and, if it become four times the natural ſize, as ſtated in

† See the figures of the lymphatics of the lower extremities, given by Mr. Cruikſhank, Fyfe, White, &c.

in the case of Phoebe Waters, then 46½ pints of matter must be added in the same, or a longer space of time, for this is not specified. Now it would be difficult to shew, that the whole of the exhalants of an inferior extremity, when acting naturally, deposit 15½ pints in it within twenty-four hours; and yet it is necessary that they should deposit twice as much in that time, according to the theory under consideration, for an accumulation of 15½ pints of lymph to take place, supposing only one-half of the lymphatic trunks of the limb to remain uninjured, because the sound lymphatics must absorb one half of the exhaled fluid, even if there were no communication betwixt them and the impervious lymphatics, by means of anastomosing branches. Hence I am led to conclude, that the exhalants do pour out an unusually large quantity of fluid into the parts affected in the first stage of Phlegmatia alba dolens puerperarum; And I conclude that the principal part of this exhaling fluid is accumulated in the cellular texture, and that only a comparatively small part is contained in the lymphatics, 1. Because I do not conceive it possible, that the whole of the lymphatics of the parts affected can contain one-third of the quantity of matter which is required to increase the bulk of the limb to the degree stated by Mr. White. 2. Because we know, from cases of pneumatosis and anasarca, that the cellular membrane of a limb is capable of being distended, either by an elastic or inelastic fluid, till the limb attains the size mentioned above. 3, Because we find it expressly stated by Zinn, in the case which he has related in the Comm. Soc. Reg. Sc. Gotting. Tom. II. *that the serum in the cellular membrane had almost assumed the nature of a tremulous jelly:* "Serum enim in tela cellulosa in gelatinæ tremulæ naturam fere abiit, omni liquidiori parte resorpta." This is the more decisive, because the limb was dissected. Various other writers have also asserted, that the accumulated matter is contained in the cellular membrane.* If the absorbents of the lower limbs should contain one half of the matter necessary for the production of the intumescence in this disease, I should expect to find many of these vessels much larger than a goose quill; in which case the operation, named *lymphée* by Fourcroy, would become almost as easy as bloodletting, in limbs affected with Phlegmatia alba dolens puerperarum: Whereas I have never found one lymphatic vessel perceptible either to the eye or touch; and am hence induced to believe, that when a perceptible enlargement of one or more of these vessels does take place, it ought to be considered as

* See Levret L'Art des Acc. § 934, Sagar. Syst. Morb. Sympt. p. 154, &c. &c.

an accidental rather than an effential circumftance, and as a confequence rather than a caufe of the complaint.

"The pain," according to Mr. White, "is occafioned by the great and fudden diftenfion of the lymphatic veffels; the whitenefs, by the parts being filled with lymph and compreffing the blood veffels fo much, that neither arteries nor veins appear externally. The tenfenefs, elafticity, hardnefs, and gloffinefs depend on the great diftenfion of the lymphatic veffels, which do not eafily give way; the uniformity of the fwelling on the diftenfion of the cutaneous lymphatics, which are innumerable." And, in page 10th of this fame work, he fays: "The great pain in the loins and in the hypogaftric region, afterwards in the conglobate glands, in the groin, ham, and the middle of the leg, and laftly in the whole lower extremity, appears to be *solely* in confequence of fudden diftenfion." It is very unfortunate for Mr. White's explanation of the manner in which pain is produced, that this fymptom *precedes* the fwelling, and is generally very much relieved by it. "*Dum partes intumescunt, dolor recedit*," fays Sauvages. Sagar, in enumerating the diagnoftic marks of this difeafe, fays it may be known, "*recessione doloris post eruptionem tumoris.*" Callifen, in one of the paragraphs quoted by Mr. White, makes this obfervation, "Surgit plerumque, 12mo. vel 14to. poft partum die tenfio & dolor inguinis, *sequitur tumor*. Nay, Mr. W. himfelf has ftated in the cafe of Elizabeth Rothwell, which I have tranfcribed*, that "fhe was attacked with pain in the groin, labium pudendi, thigh and leg of the fame fide, *which swelled, and then the pain abated*." Now an effect cannot precede its caufe, and we muft therefore reject Mr. White's explanation of the production of that pain which occurs at the very commencement of this difeafe. The pain which afterwards arifes from, or is kept up by, the diftenfion of the fkin, is of a much lefs excruciating kind. It will be a fufficient reply to the latter part of the above extract, that Mr. White has completely failed to fhew, that the accumulated lymph is contained in the abforbents. If the cutaneous lymphatics were really as much diftended as he has reprefented, we fhould expect to find the fkin lefs white than it is found to be; veffels with tranfparent coats, and containing a tranfparent yellowifh fluid, cannot impart to the fkin a whitenefs which they do not poffefs themfelves.

"By this great diftenfion," fays Mr. White, "and confequent compreffion, the exhalants are prevented from fecreting fo

* See above in page 136.

fo much lymph, and confequently there is not fo great a fupply. The lymphatic glands fometimes grow painful and fwell, which is owing to the *vasa inferentia* fending the lymph into them quicker than the *vasa efferentia* can difcharge it. Pains will fometimes attack parts, which have neither lymphatic glands, large nerves, blood veffels, nor lymphatics, which can only be accounted for from fympathy." The enormous effufion of fluid into the cellular texture of the limb appears to be checked partly by the diminifhed action of the arteries in confequence of this topical evacuation of them, and partly by the refiftance which the diftenfion of the fkin prefents. The painful enlargement of the lymphatic glands, which fometimes takes place in the courfe of the difeafe under confideration may, I conceive, be much more fatisfactorily afcribed to inflammation, communicated to them from the parts in which they are imbedded, or excited in them by the abforbed fluid. When the enlargement and induration have taken place, the paffage of the lymph into them will, in all probability, be obftructed, and the quantity that actually enters them diminifhed. There appears to be abundant caufe for idiopathic pain in every part affected with Phlegmatia alba dolens puerperarum, fo that we have no occafion to recur to fympathy for an explanation; and I muft confefs myfelf ignorant of any pains attacking parts, "which have neither lymphatic glands, large nerves, *blood-vessels*, nor *lymphatics*."

Mr. White next informs us, that "the words *calidus*, hot or warm, when fpeaking of the fwelling of the limb, are made ufe of in contradiftinction to *leucophlegmatia*, in which the limb is white and cold; in this diforder it is white and warm. When fpeaking of increafed heat, it is to be underftood of the whole body, not of the limb alone, as that does not appear to be hotter than the other parts of the body." In the general hiftory of this difeafe, given in his former publication, Mr. W. fays, the limb "is *hot*, and exquifitely tender," p. 8; and a degree of heat greater than is expreffed by the term *warm*, is, in my opinion, meant by all the writers on this malady. Hitherto I have never taken the temperature of either the body or limb by a thermometer, which I regret: But, as far as I have been able to judge from the feel, the affected limb is generally hotter than the found one, and the whole body hotter than natural in the firft ftage of the diforder. To the fenfation of the patient likewife the difordered limb has, in various inftances, been hotter than the reft of the body, and the pain is by fome patients reprefented as of a burning kind.

"There is heat in all fevers," continues Mr. W. "but that does not imply inflammation, every fever is not inflammatory. There is a quick pulfe, but that is owing to irritation by the

ſudden and violent diſtenſion of the irritable coats of the lymphatic veſſels." In his former publication, we are expreſsly told, that there is inflammation in the firſt ſtage of the diſeaſe. He ſays, "but as this *inflammation* is not the original diſeaſe, but a ſymptom only, &c. &c. p. 59. Whether the inflammation be the primary or ſecondary complaint, I ſhall have occaſion to conſider hereafter, when I come to defend my own theory; with reſpect to the quickneſs of the pulſe, Mr. W. ſeems to forget, that rigors and frequent pulſe, &c. not uncommonly precede the pain; and as it has been ſhewn above, that the pain precedes the ſwelling, we cannot with any degree of propriety attribute the quick pulſe to the ſudden and violent diſtenſion of the lymphatics.

Mr. White, in the next place, ſays, "if you puncture the ſkin with a lancet, the lymph does not flow out as in anaſarca, where it is thin, and is lodged in the cells of the cellular membrane, which communicate throughout the whole body. In this diſorder, you do not puncture the trunks of the abſorbents, but the *minima vasa* only of the cutaneous lymphatics. The violent pain and diſtenſion do not continue many days; *the anastomosing lymphatics begin to enlarge*, and by degrees carry off the obſtructed lymph; but it is many weeks before it has obtained a perfectly free paſſage." It has been ſufficiently demonſtrated already, that the intumeſcence of the limb is principally, if not ſolely, occaſioned by the effuſion of a fluid into the cellular membrane, which ſpeedily coagulates. Hence, when a puncture, or ſmall inciſion is made through the ſkin early in the complaint, little, if any, fluid iſſues out: But afterwards, as the affection of the limb approaches more to anaſarca, a greater diſcharge of liquid iſſues from a puncture of the ſkin, becauſe the proportion of fluid to the coagulum in the cellular membrane is gradually increaſing, as is explained in my Eſſay on Phlegmatia dolens, p. 208. If the lymph be collected in the lymphatics, as is ſtated by Mr. W. though only the vaſa minima ſhould be punctured, I ſhould expect a conſiderable flow of lymph from them; for when the ſmall arteries or veins of a part are enlarged, theſe, when punctured, bleed freely. Mr. White ſeems to admit, that the intumeſcence of the limb is occaſioned by the effuſion of fluid into the cellular membrane in the laſt ſtage of the diſeaſe: For he ſays, in page 80, "whenever the ſwelling of the limb loſes its elaſticity, and pits upon preſſure, it is probable that many of the lymphatic veſſels burſt." Now, if the lymphatics do burſt, their fluid muſt of courſe paſs into the cellular membrane of the limb; but if the lymphatics ſhould burſt, we might expect the inflammatory ſtage of the diſeaſe to be reproduced according to his theory, when theſe ruptured lymphatics lymphatics became healed, which is not agreeable to facts. It appears very extraordinary to me, that the lymphatics of an inferior extremity should be supposed to be so much distended, as to contain from 15 ½ to 46 ½ pints of lymph in addition to their natural quantity, and *that the anastomosing vessels should then only be beginning to enlarge*. Why does not the enlargement of the anastomosing branches keep pace with that of the other branches of the lymphatics? And why does not the disease cease as soon as these anastomosing branches are so far increased, as to be able to transmit the quantity of lymph formerly conveyed by the trunks supposed to be obstructed, and long before the swelling has arrived at half the bulk which we find it attains? Three or four lymphatic trunks, of one-twelfth of an inch in diameter, are, perhaps, all that can be divided in the situation which Mr. White has fixed upon: Therefore, according to his hypothesis, as soon as the anastomosing branches of the limb are so far enlarged, as to be able to transmit as much lymph as these three or four trunks can, which are supposed to be obstructed, the complaint should begin to subside and go off. This must happen, I conceive, long before the lymphatics of the limb can be distended to that extreme degree which will enable them to contain the quantity of lymph mentioned above. How inconsiderable an augmentation of each of the numerous anastomosing branches must be sufficient to convey every particle of lymph that should have passed through the trunks supposed to be obstructed, to those that are uninjured and pervious!

Mr. White concludes his recapitulation, by saying, "this disorder has not been known to return in the same limb, though women have had several children after having had this complaint; because the same accident cannot happen a second time to those lymphatics.

The first case which I saw, affected both the lower extremities of a lady who was lying-in, very severely, and it was the second attack. She was unable to suckle her child, and attributed it to the milk settling there. She was not alarmed by the complaint, and knew how to manage it better than I did at that time. Mr. White may, however, reject this case as not a genuine case of his Phlegmatia alba dolens puerperarum, because I neglected inquiring into the state of the labia pudendi, &c. As she is now dead, I cannot obtain the necessary information on this point. If nothing more could be adduced against Mr. White's theory than the recurrence of the disease in the same limb, I do not see how this circumstance could be urged against it; for we know that the intumescence of the limb is much greater in some patients than others. Mr. White's own cases cafes teach us, that the limb becomes fometimes double, and fometimes four times the natural fize. Now, the eafieft mode of accounting for this, agreeably to Mr. White's hypothefis, would be to fuppofe, that one half only of the lymphatics which pafs over the fharp ridge of the os pubis, and defcend into the pelvis, is divided in the former cafe, and the whole in the latter cafe; confequently the fame limb may be fuppofed to have one half of thefe lymphatics divided in one labour, and the remaining half in a fucceeding labour, which will account for the production of the difeafe twice in the fame limb. Further, we may conceive, agreeably to the fame hypothefis, that in one cafe an inferior extremity may become tumefied without any affection of the labium pudendi of the correfponding fide; that in another cafe the intumefcence of one extremity may be accompanied by an affection of both labia pudendi; that in a third cafe, one or both of the labia pudendi may become tumefied, without any fwelling of the lower extremities; according as one or more points of the fharp ridges of the offa pubis have been preffed open by the fetal head during labour.

When Mr. White wrote the firft part of his Inquiry, he had not known Phlegmatia alba dolens puerperarum to happen "to a woman more than once, though fhe has afterwards had more children," p. 11: But, in the fecond part, he mentions a lady who had the difeafe on one fide in her firft labour; and "in her fecond confinement fhe had it on the other fide," p. 87. Again, in the former part of his Inquiry, we find him afferting, that this difeafe "never comes to fuppuration," page 38: In the fecond part he relates a cafe, where an abcefs formed and burft; and endeavours to explain why it formed, and alfo fome other circumftances relative to it. See pages 113, &c. After thefe occurrences, I fhould not have expected to find Mr. White confidently afferting in the extract, upon which I am commenting, that the fame accident cannot happen a fecond time to the lymphatics, which pafs over the os pubis and defcend into the pelvis, and declaring in a former part of the work (page 49), that a cafe, which I faw and have related in my Effay on Phlegmatia dolens, "had fymptoms which are not compatible with this diforder." Mr. White may, perhaps, very properly affirm, that he has not yet feen the difeafe occurring twice in the fame limb, and that he has not witneffed the fymptom or fymptoms, which he deems incompatible with the diforder: But I am convinced, that he is neither juftified in faying, that the fame limb cannot be twice affected; nor in affirming, that the cafe juft alluded to, had fymptoms which are not compatible with this diforder. We fhould be cautious in declaring any any ſymptom incompatible with a diſeaſe which does not imply a contradiction; and may properly ſay with *Demea:*

"Nunquam ita quiſquam bene ſubducta ratione ad vitam fuit,

Quin res ætas, uſus, ſemper aliquid adportet novi,

Aliquid moneat, ut illa, quæ te ſcire credas, neſcias:

Et quæ tibi putaris prima, in experiundo repudies."

TERENT. ADELPH. Act. V. Sc. 4.

[ To be continued. ]

